# Heterogeneous environments shape invader impacts: integrating environmental, structural and functional effects by isoscapes and remote sensing

**DOI:** 10.1038/s41598-017-04480-4

**Published:** 2017-06-23

**Authors:** Christine Hellmann, André Große-Stoltenberg, Jan Thiele, Jens Oldeland, Christiane Werner

**Affiliations:** 1 Ecosystem Physiology, University of Freiburg, Georges-Köhler-Allee 53/54, 79110 Freiburg, Germany; 2 Experimental and Systems Ecology, University of Bielefeld, Universitätsstraße 25, 33615 Bielefeld, Germany; 3 Institute of Landscape Ecology, University of Münster, Heisenbergstraße 2, 48149 Münster, Germany; 4 Biodiversity, Ecology and Evolution of Plants, Biocentre Klein Flottbek and Botanical Garden, University of Hamburg, Ohnhorststraße 18, 22609 Hamburg, Germany

## Abstract

Spatial heterogeneity of ecosystems crucially influences plant performance, while in return plant feedbacks on their environment may increase heterogeneous patterns. This is of particular relevance for exotic plant invaders that transform native ecosystems, yet, approaches integrating geospatial information of environmental heterogeneity and plant-plant interaction are lacking. Here, we combined remotely sensed information of site topography and vegetation cover with a functional tracer of the N cycle, δ^15^N. Based on the case study of the invasion of an N_2_-fixing acacia in a nutrient-poor dune ecosystem, we present the first model that can successfully predict (*R* ^2^ = 0.6) small-scale spatial variation of foliar δ^15^N in a non-fixing native species from observed geospatial data. Thereby, the generalized additive mixed model revealed modulating effects of heterogeneous environments on invader impacts. Hence, linking remote sensing techniques with tracers of biological processes will advance our understanding of the dynamics and functioning of spatially structured heterogeneous systems from small to large spatial scales.

## Introduction

Environmental heterogeneity is a key factor structuring plant communities^1–3^. While spatial heterogeneity determines plant performance, plants in turn feed back on their environment, thereby affecting multiple factors such as abiotic conditions, nutrient cycling or soil biota^4, 5^, thus further increasing spatial heterogeneity. Abiotic conditions can modulate the outcome of plant-plant interactions^6^, which is reflected in changes in the balance between competition and facilitation along environmental gradients (e.g. refs 7–9). However, there is a clear lack of knowledge and quantitative assessments in how far small-scale heterogeneity within plant communities affects the outcome of plant-plant interactions. In fact, although the effects of competition and facilitation clearly must be considered to be spatially dependent^10^, the spatial range in which these interactions take place is largely unknown^11, 12^. This is particularly relevant for prediction of invasion impacts, as plant invasion rarely occurs evenly distributed in recipient communities, particularly during early stages of invasion. Many theories, such as the intermediate disturbance hypothesis^13^, fluctuating resource hypothesis^14^, concepts of invasibility of recipient community^15^ or niche opportunities^16^ all point towards the importance of the interplay between invader and native species interaction and local environment conditions. However, to what extend environmental heterogeneity modulates these interactions is far from being resolved. Thus, there is a clear need for methods which can adequately trace functional changes and plant-plant interaction spatially at the community scale. In particular, novel tools and approaches are required that integrate geospatial information on functional tracers in heterogeneous environments.

Stable isotope ratios could provide such functional tracer, as they reflect ecological and physiological processes across temporal and spatial scales^17–19^. Spatially explicit representations of isotopic signatures, termed *isoscapes* ^20^, provide novel tools to resolve processes at the community scale^12, 21–23^. Specifically, the stable isotopic composition of nitrogen, δ^15^N, provides a sensitive tracer of changes in the N cycle^24–26^, or of different N sources within plant communities^27–29^. Applied in a spatially explicit manner, δ^15^N isoscapes revealed the spatial extent of N input by an exotic N_2_-fixing plant invader into the invaded plant community^12, 21, 30^. Symbiotically fixed N has an isotopic signature that differs from soil N-sources in many systems, the former being close to the atmospheric value of 0‰. In ecosystem with low N availability δ^15^N of non-fixing native species can be markedly depleted. Therefore, an increase in δ^15^N of their foliage in the vicinity of the N_2_-fixing invader (approaching δ^15^N of 0‰) indicates significant uptake of fixed nitrogen derived from the invader.

Yet, detecting spatial patterns of functional changes is often complicated in the presence of system-immanent spatial heterogeneity. Accordingly, establishing spatially explicit predictive models of plant functional tracers such as foliar δ^15^N is challenging due to the complexity and spatial variability of different processes. In the case of N-cycling, such processes can be e.g. transformation of nitrogen within or N-losses from terrestrial ecosystems^31, 32^. To date, integrated approaches which can incorporate such spatial variability of heterogeneous environments as well as influences of interacting plants are still lacking.

Linking remote sensing with functional tracers can provide a novel way to include spatially dependent processes in community-scale models. For instance, airborne LiDAR (light detection and ranging) yields spatially explicit data that can capture land-surface attributes, such as site topography or vegetation cover^33^, which potentially govern ecosystem processes. Topography is a major control of physical soil conditions, often correlating with N transformation rates^34, 35^. Similarly, vegetation cover can modify physical conditions of the soil, such as humidity and temperature, as well as organic matter content, nutrient availability and microbial activity^4, 36, 37^. In turn, these factors can influence N cycling and therefore, δ^15^N values of plants^24, 38, 39^. Accordingly, landforms and associated soil moisture patterns^35, 40, 41^, as well as vegetation cover^35, 42^ have previously been shown to correlate with plant or soil δ^15^N. This indicates that variation of δ^15^N in heterogeneous landscapes can potentially be predicted from LiDAR-derived land-surface attributes, thereby allowing us to disentangle spatial influences of invasive species on native species δ^15^N from background variation.

**Figure 1:**
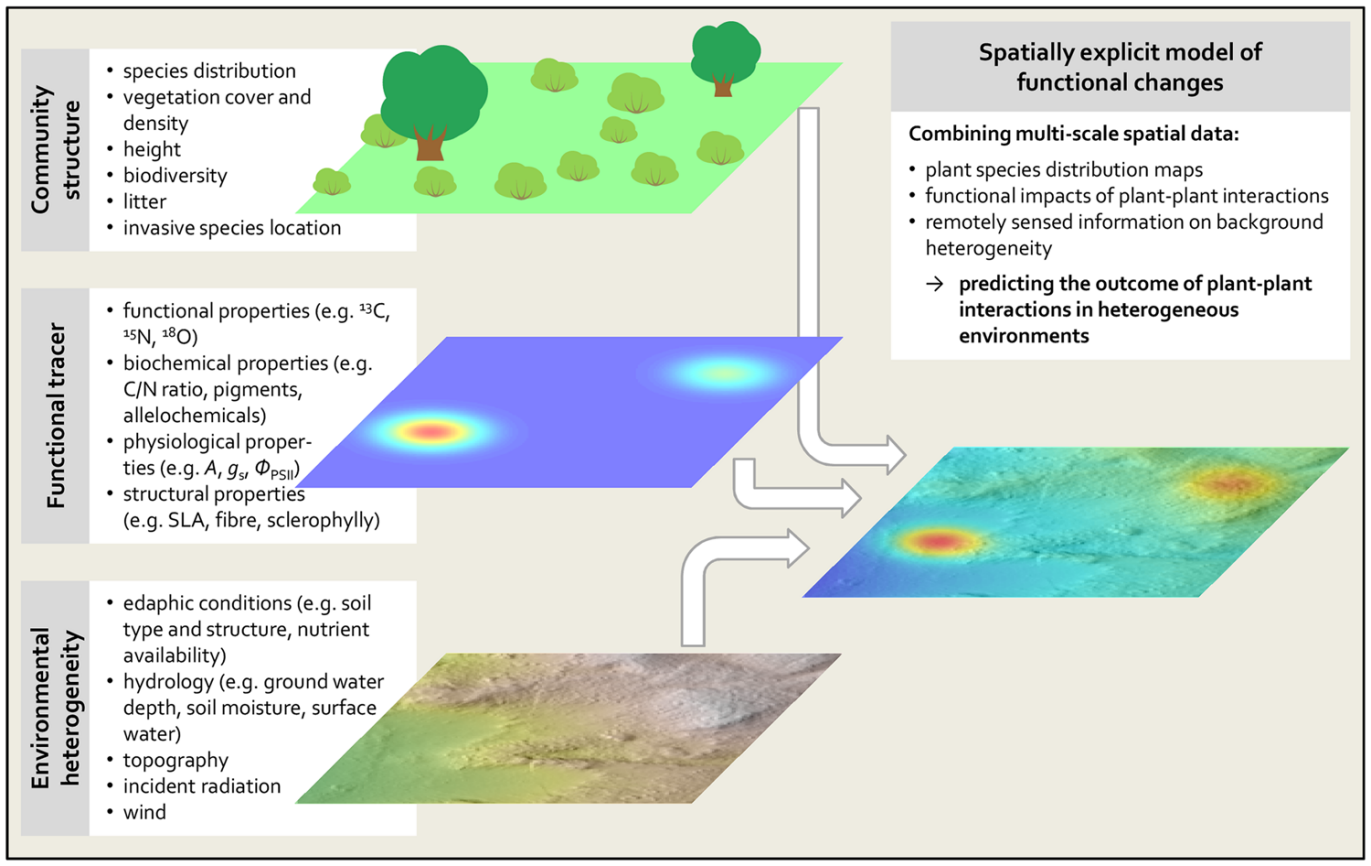
Schematic illustrating how different layers of spatial information can be combined into one joint model to yield spatially explicit predictions of functional tracers. Potential predictors can include the distribution of key species (e.g. presence of an invasive plant species), structural information, or raster maps of abiotic environmental conditions. Data can be derived from field measurements or from remote sensing products. The functional tracer can, for example, be measured in the vegetation or in the soil and should be suitable for spatially explicit data acquisition. *A* – rate of photosynthetic carbon assimilation; *g*_*s*_ – stomatal conductance; *Φ*_PSII_ – maximum quantum efficiency of photosystem II; SLA – specific leaf area.

Here, we combine multi-scale and multi-source georeferenced spatial data into one joint model. We propose that integrating spatial mapping of plant species, functional tracers of plant-plant interactions and remotely sensed information on spatial variability within ecosystems can give new insights into the significance of environmental heterogeneity for the outcome of plant-plant interactions and for community functioning. The conceptual framework of this approach is outlined in Fig. 1.

To test this framework, we modelled the spatial pattern of foliar δ^15^N as a functional tracer of the impact of an N_2_-fixing invasive species in a Mediterranean dune ecosystem. We hypothesized (1) that both, the presence of an N_2_-fixing plant invader as well as the inherent, spatially heterogeneous properties of the native system, i.e., site topography and vegetation cover, are important variables affecting δ^15^N of a non-fixing native species, (2) that using these variables as spatially explicit predictors, a model can be created that allows to predict spatial patterns of δ^15^N isoscapes on community scale, and (3) that such a model can reveal interactions between environmental heterogeneity and the outcome and spatial scales of plant-plant interactions.

## Results

**Figure 2:**
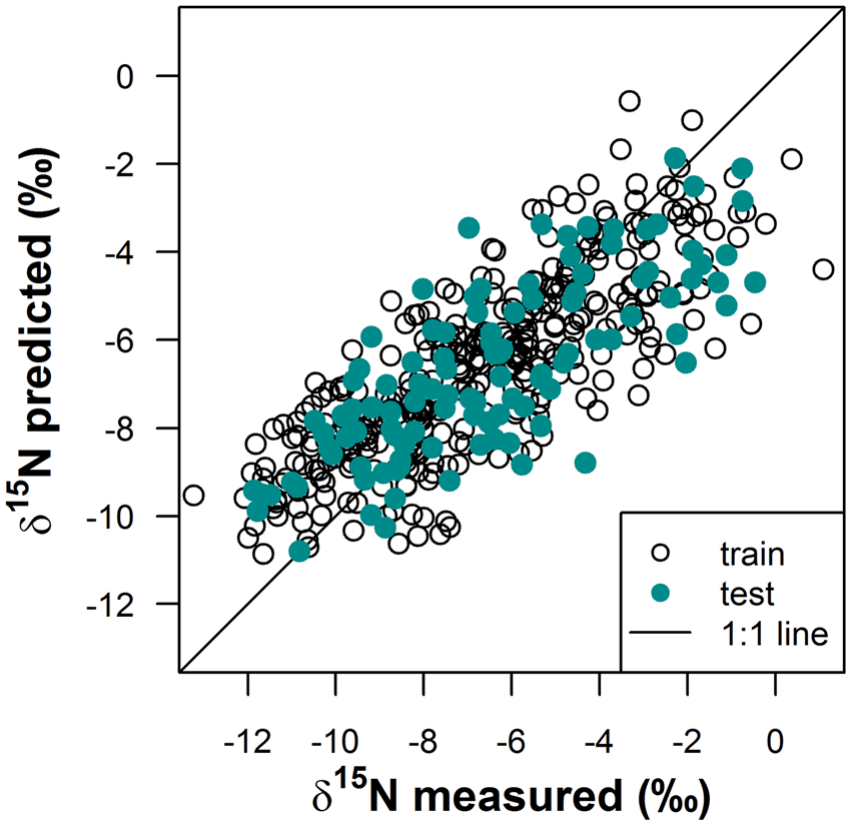
Measured foliar δ^15^N values of *C. album* versus δ^15^N values predicted by the additive mixed model for the training and the test set of the model which was closest to the median *R* ^2^ out of 100 model runs with random data splits. The 1:1-line is indicated.

Foliar δ^15^N of *Corema album*, as a functional tracer of N cycling and N input, showed an extraordinarily large range between −13.2 and 1.1‰ in the heterogeneous dune ecosystem, while δ^15^N of the N_2_-fixing *Acacia longifolia* was close to the atmospheric value of 0‰ with little variation (−1.6 ± 0.46‰, mean ± SD). The strong variation in *C. album* δ^15^N was modelled effectively using a generalized additive mixed model (GAMM), with remotely sensed attributes (such as topography and vegetation cover) and spatial relationships between individual plants (i.e. distance and elevation relative to the invader) as predictors. The model showed a good predictive ability as demonstrated by a good agreement between measured and predicted values (Fig. 2). The median *R* ^2^ was 0.6 (interquartile range (IQR): 0.57–0.64) and median RMSE was 1.82‰ (IQR: 1.72–1.89), as calculated based on test sets from 100 model runs with random data splits between training and test sets.

**Figure 3:**
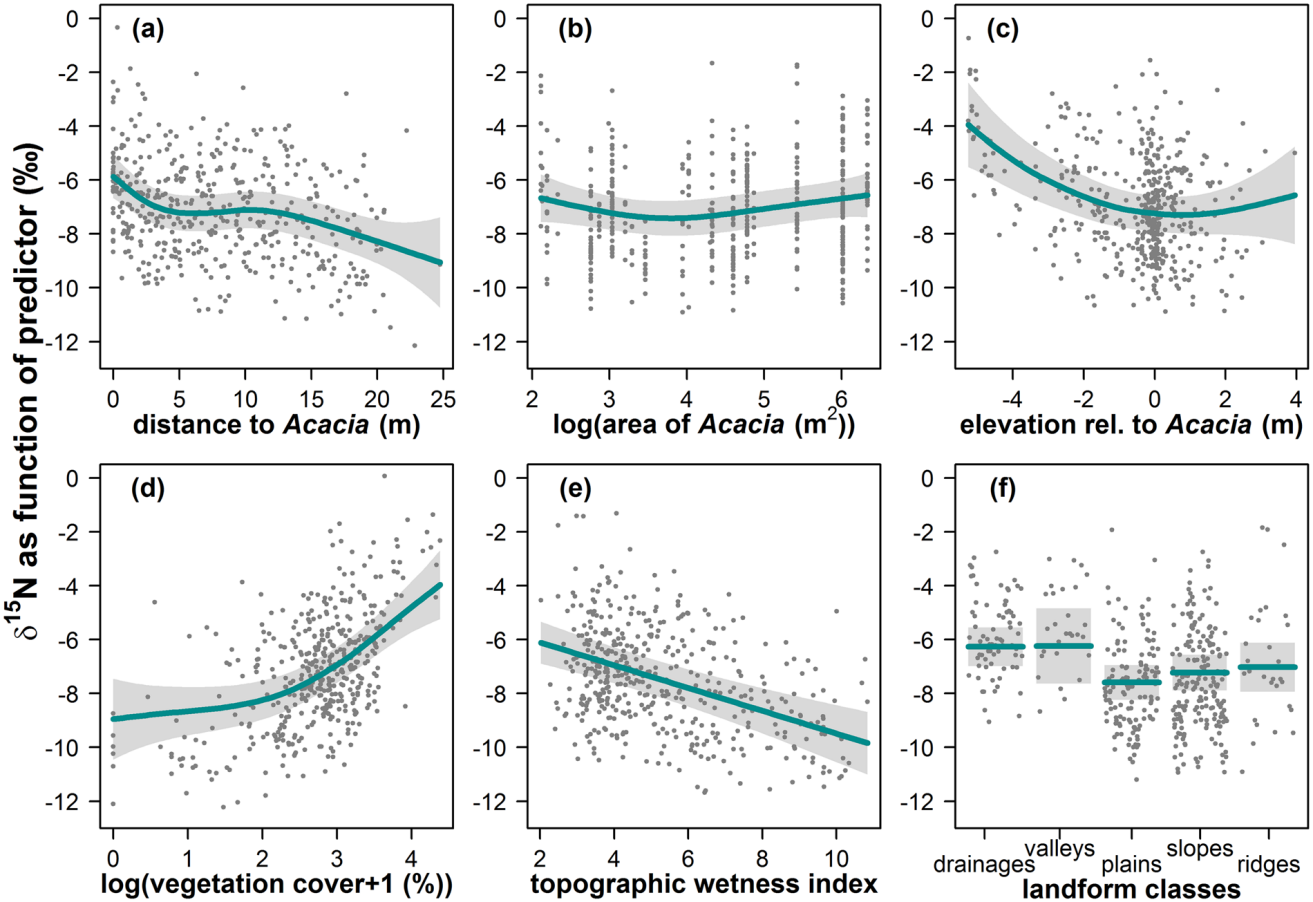
(**a–e**) Smoothing functions used by the generalized additive mixed model. Smoothers describe δ^15^N as a function of the respective predictor (lines) along with their 95% confidence intervals (grey shading) and partial residuals (points), which are the residuals for each predictor when all other predictors are held constant at their mean (continuous predictors) or at the most frequent class (for factors). (**f**) Effect of the categorical predictor landform classes.

In the model selection, the best model with an AIC_c_ weight of 0.73 included all fixed effects with the exception of the canopy extent of *A. longifolia*, while the second best model (AIC_c_ weight = 0.24) comprised the complete set of predictors (Table 1). For the distance to *A. longifolia*, the GAMM predicted an enrichment of δ^15^N by *ca*. 3‰ close to the canopy of the invader, with decreasing δ^15^N the larger the distance to the canopy (Fig. 3a). This relationship was highly non-linear (effective degrees of freedom = 4.36), with ^15^N enrichment being evident in a range of approximately 5–8 m from the canopy. The area of the *A. longifolia* canopies ranged from 8.3 to 564.7 m^2^. The smoother for the logarithm of the canopy area showed no clear patterns for the smallest *A. longifolia* individuals, but indicated a weak trend towards ^15^N enrichment in the neighbourhood of very large canopies, which accounted for approximately 1‰ increase (Fig. 3b). The elevation of *C. album* plants relative to *A. longifolia* was most important for individuals that grew downslope of *A. longifolia* (Fig. 3c). These plants were considerably ^15^N enriched compared to individuals growing at level with or above *A. longifolia*. Vegetation cover varied strongly across and within the study sites. While most *C. album* individuals grew at locations with sparse vegetation cover below 40%, patches with bare sand (0%) and with very dense vegetation (92%) also occurred. Vegetation cover accounted for substantial enrichment in *C. album* foliar δ^15^N of up to ca. 5‰ in the densest compared to the sparsest patches in this study (Fig. 3d). Furthermore, δ^15^N decreased linearly by ca. 4‰ with increasing topographic wetness index (TWI, Fig. 3e). Regarding the landform classification, valleys and drainages were most enriched in δ^15^N while plains were the most depleted landform class (Fig. 3f).

**Table 1:** Results of a model selection procedure, ranked by AICc, showing the four models with strongest support.

*Intercept*	*Landform*	*s (dist.Ac)*	*s (elev.Ac)*	*s (log(area.Ac)*	*s (log(cover))*	*s (TWI)*	*df*	*logLik*	*AICc*	*delta*	*weight*
−5.84	+	+	+		+	+	16	−840.2	1713.7	0.0	0.73
−5.79	+	+	+	+	+	+	18	−839.2	1715.9	2.2	0.24
−5.78	+	+			+	+	14	−845.7	1720.4	6.7	0.03
−5.71	+	+		+	+	+	16	−844.8	1722.8	9.2	0.01

A plus sign indicates the inclusion of the smoother (s()) or the categorical predictor (landform class), respectively, in the candidate model. *dist.Ac*, *elev.Ac*, *area.Ac* – distance to, elevation relative to, and area of the closest *Acacia longifolia* canopy; *cover* – vegetation cover+1, *TWI* – topographic wetness index; *logLik* – log likelihood; *AIC*_*c*_ - Akaike Information Criterion with finite sample correction; *delta* – difference in AIC_c_ with respect to the lowest AIC_c_; *weight* – AIC_c_ weight (corresponds to the conditional probability of each candidate model).

Supplementary Fig. S1 shows the predictions for the individual transects, using only the fixed effects. The predicted values fitted the measured data in many cases, while notably, even where deviations (due to random effects) were evident, in general the model still succeeded to reproduce the general trend in the data (Supplementary Fig. S1).

**Figure 4:**
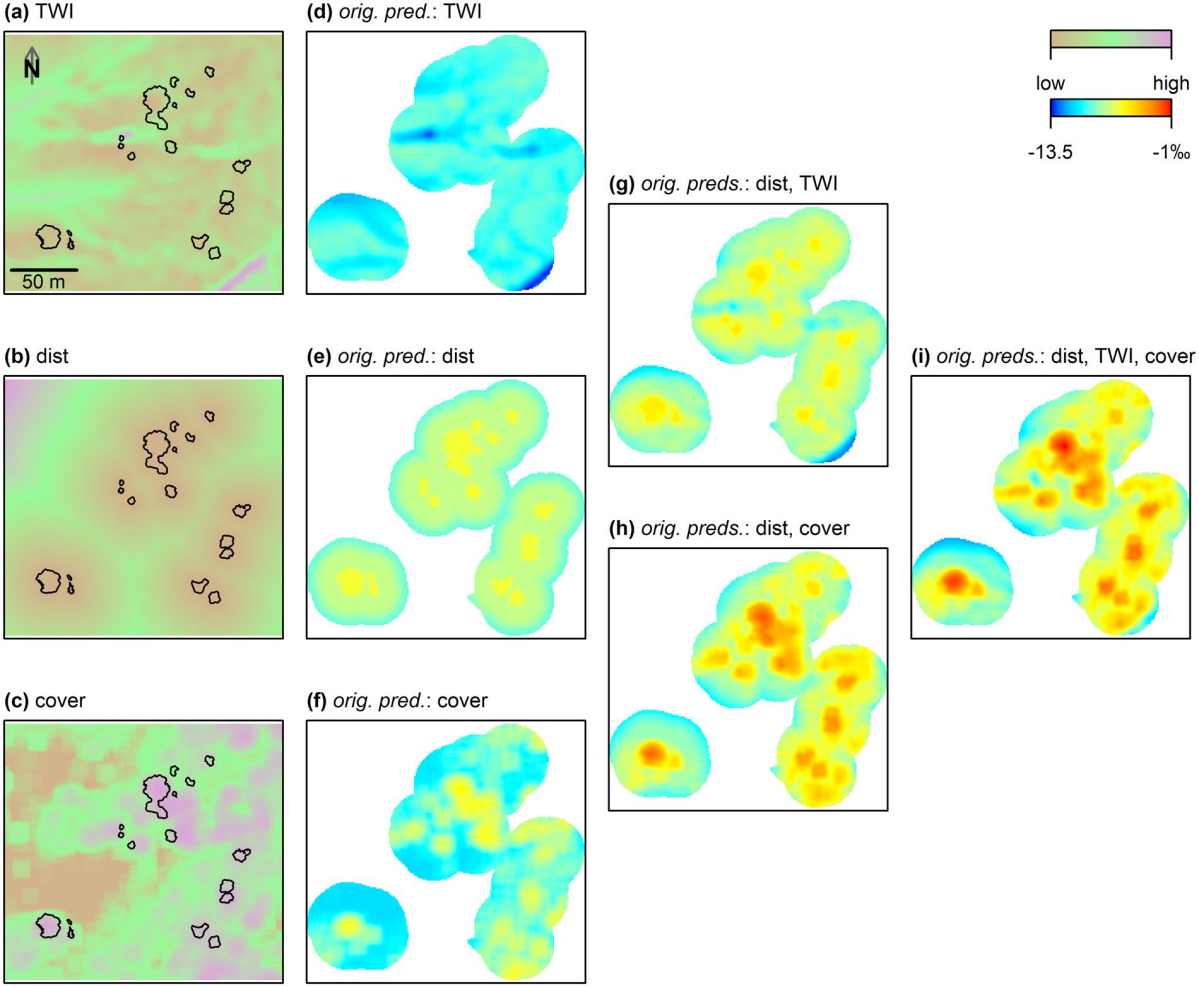
Model simulations illustrating the additive effects of three selected spatially explicit predictors on model estimates for the study site Pinheiro da Cruz. (**a–c**) Raster maps of the predictors TWI (topographic wetness index), *dist* (distance to the closest *Acacia longifolia* canopy) and *cover* (log(vegetation cover +1)) are shown. Black outlines demarcate *A*. *longifolia* canopies. (**d–i**) Model simulations of *Corema album* foliar δ^15^N using a generalized additive mixed model (GAMM) with six predictors (including TWI, *dist* and *cover*, see Methods section for details). Predictors specified in the panel heading are included with their original spatial variation while all other predictors are held constant at their mean (for continuous predictors) or at the most frequent class (for factors). First, only one predictor was used with its original variation, with all other variables held constant (**d–f**), second, additive effects of two original predictors are illustrated (**g,h**), and finally, (**i**) shows the combined effect when taking into account the spatial variation of three predictors.

With the validated model and all of the predictors being available as raster maps, it was then possible to create predictive isoscapes for the surroundings of mapped *A. longifolia* individuals. As an example, Fig. 4 illustrates the additive effects of three important predictors (topographic wetness index, distance to *A. longifolia*, and vegetation cover) for one of the study sites (Pinheiro da Cruz; see Supplementary Figs S2–S5 for the other sites). Considering solely the spatial variation of one predictor at a time, while keeping all others constant, indicates that all predictors affected the predictions of *C. album* foliar δ^15^N, but to different degrees and resulting in different spatial patterns (Fig. 4d–f). While the isolated effect of *A. longifolia* presence created a regular pattern surrounding its canopies, the combination with the topographic wetness index (Fig. 4g) or with vegetation cover (Fig. 4h) considerably increased the range and the patchiness of δ^15^N predictions. The interplay of all three variables (Fig. 4i) generated the largest spatial variation, with highest predicted δ^15^N values at locations where proximity to *A. longifolia*, low topographic wetness index and high vegetation cover coincided (Fig. 4g–i). Particularly strong depletion in δ^15^N was evident at locations where the topographic wetness index was high (Fig. 4d,g,i).

**Figure 5:**
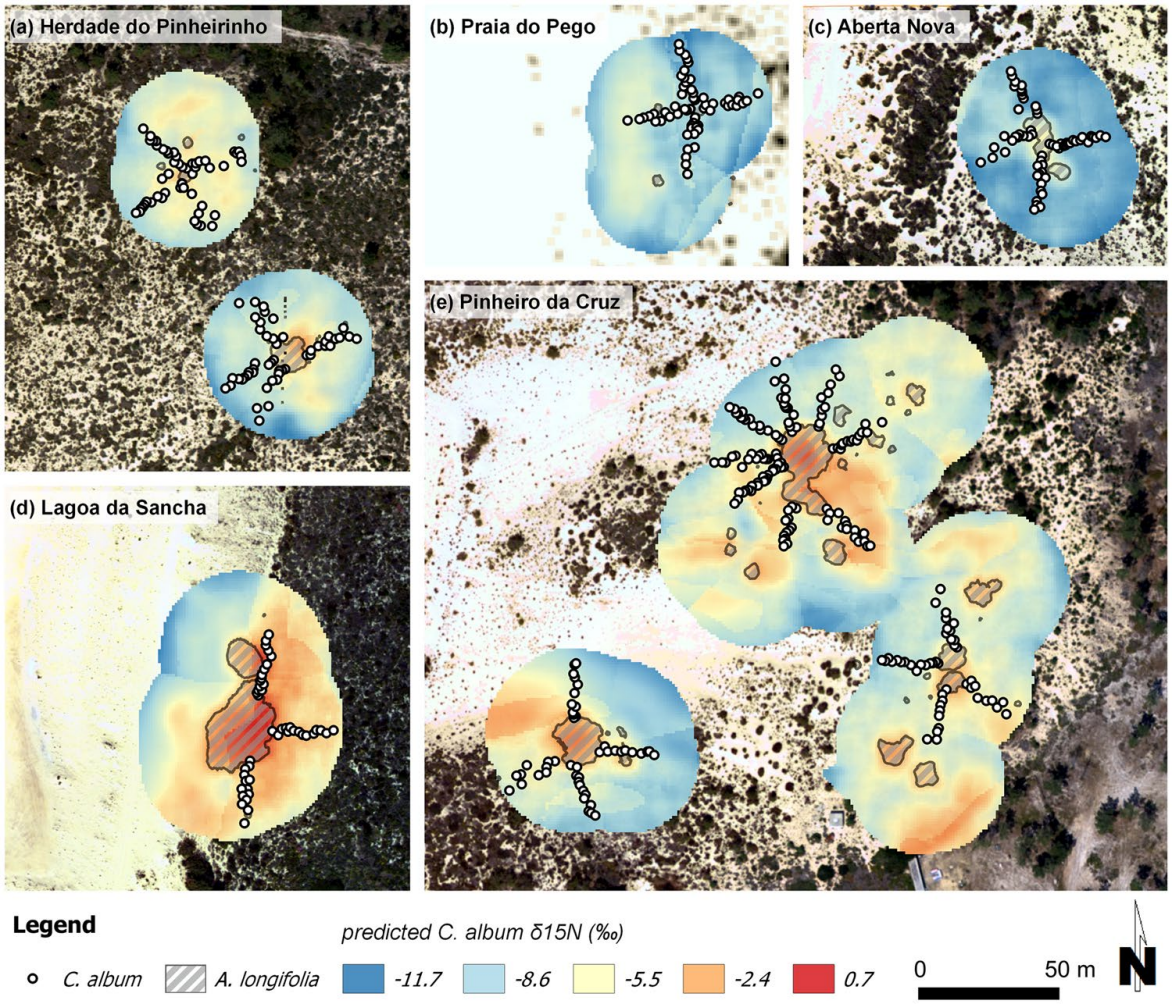
(**a–e**) Predicted δ^15^N isoscapes for *Corema album* foliage at the five study sites. *Acacia longifolia* canopies (grey hatching) and sampled transects of *C. album* (points) are indicated. In the background, aerial images of the study sites are shown with the exception of (**b**), where no high-resolution aerial image was available and vegetation cover is displayed instead.

Figure 5 shows predicted δ^15^N isoscapes for all study sites, using the complete set of predictors with their original spatial variation. The isoscapes revealed considerable variation of δ^15^N at small spatial scales, with changes of up to 5‰ at a distance of 10 m (Fig. 5). At the different sites, the prominent effect of vegetation cover in the native system was most obvious. For instance, at Lagoa da Sancha, a sharp change between bare sand and dense vegetation caused a strong and rapid enrichment in predicted δ^15^N (Fig. 5e), while values were generally very depleted wherever vegetation cover was low. Enriched δ^15^N values were found close to *A. longifolia* canopies, with particularly high enrichment where *A. longifolia* presence concurred with high vegetation cover (Fig. 5d,e). Furthermore, even small individuals of the invader occasionally affected extended areas that stretched downslope, as for example in Praia do Pego and Pinheiro da Cruz (Fig. 5b,e). This reflected the interaction between site topography and presence of *A. longifolia*, which indicated the strong effect on δ^15^N of *C. album* individuals growing down-slope of the invader (Fig. 3c). Hence, the modelling results suggested that strong spatial variation of foliar δ^15^N was the integrated result of the invader impact and the distinct spatial patterns of heterogeneous environmental factors.

## Discussion

Combining spatially explicit information of environmental, structural and functional effects into one joint community-scale model offers new perspectives to advance our understanding of the dynamics of plant-plant interactions in heterogeneous plant-soil systems. Specifically, using stable isotopes as powerful tracers, field-scale isoscape models can add a novel dimension to community ecology^43^. Here, by taking the invasion of an N_2_-fixing invasive acacia in a nutrient-poor dune ecosystem as an example, we present the first model that can predict small-scale spatial variation of foliar δ^15^N, as a spatial tracer of N cycling and N input, from observed geospatial data. Modelling ^15^N isoscapes of a non-fixing native species as a function of vegetation cover, variables related to site topography and locations of the invasive *A. longifolia* revealed modulating effects of heterogeneous environments on invader impact. This clearly emphasizes that the influence of an invasive species has to be considered within its spatial context. In fact, impact assessment may be biased when processes potentially interacting with invader influence – and their spatial dimension – are not accounted for.

The model predicted foliar δ^15^N with high precision and accuracy (Fig. 2) and effectively retraced pronounced small scale patterns along transects in the heterogeneous environment (Supplementary Fig. S1). Our approach demonstrated that the large spatial variation in the functional tracer can be explained by the interplay of plant-plant interactions and patchy environmental conditions (Figs 3 and 4). We identified three main controls of δ^15^N in the native system: vegetation cover, topographic wetness index and landform class. Generally, δ^15^N of ecosystems can be influenced by changes in the balance of N input vs. output and fractionation, with potentially large effects of N losing processes^24, 38, 39^. As increasing rates of N transformations and N losses are typically linked to soil moisture, and as topography is an important control of hydrological processes, correlations between topographic position and δ^15^N of non-fixing plants across landscapes in different ecosystems have been attributed to moisture-related patterns of mineralization, nitrification and denitrification (e.g. refs 35, 40 and 41). In our study, we used a landform classification to characterize the topographic position relative to its surrounding for each individual plant as well as a topographic wetness index as a proxy for soil moisture. In line with the literature, plants growing in drainages and valleys showed more enriched δ^15^N values compared to plants from plains, slopes and ridges (Fig. 3f). However, according to the topographic wetness index, moisture – as affected by topography – apparently was not the driving factor in enriching δ^15^N. In contrast, we found a negative relationship between the wetness index and δ^15^N (Fig. 3e), indicating a decoupling of the effects of landform and topographically controlled moisture in this well-drained sand dune ecosystem. Here, foliar δ^15^N could be affected by landform class, e.g., via relationships with soil texture and organic matter content^22^ or plant litter accumulation in topographic depressions^44^.

The strong negative relationship between the topographic wetness index and δ^15^N in our study (Fig. 3e) cannot be explained by altered rates of N transformations and N loss, as these would typically increase with increasing soil moisture and lead to enrichment of residual δ^15^N. Beside N-transformation processes, mycorrhizal association is known to strongly affect plant δ^15^N values, with ericoid mycorrhiza potentially causing substantial depletion^38, 45^, and the influence may additionally be modulated by N availability^45, 46^. As mycorrhizal fungi are considered particularly influential in nutrient limited systems^28^, and for species without permanent access to ground water as *C. album* ^47^, their impact in this dune ecosystem can be expected to be large and variable within the heterogeneous environment. Thus, one hypothesis explaining the influence of the topographic wetness index could be that soil moisture affects foliar δ^15^N by controlling fungal N retention or N supply to the plant.

Vegetation cover accounted for high variation in δ^15^N of up to 5‰ between the lowest and the highest cover values, with strong δ^15^N enrichment at densely covered sites (Fig. 3d). A comparable enriching effect of vegetation cover on foliar δ^15^N was also found in Brazilian white-sand vegetation^42^ and, based on NDVI, in a semi-arid ecosystem^35^. Vegetation cover and root systems modify physical conditions in the soil, such as humidity and temperature, as well as organic matter content, nutrient availability and microbial activity^4, 36, 37, 48^. Therefore, patchy vegetation can increase spatial heterogeneity through multiple feedbacks on soil properties and processes, such as protection from erosion, higher soil infiltration and water-holding capacity, larger soil C and N pools and higher microbial activity as well as by constituting sinks for runoff, sediment and nutrients (ref. 35 and references therein).

In conjunction, landform class, topographically controlled wetness and vegetation cover create a mosaic of microsites where the activity of N transforming processes and the effects of mycorrhiza may vary significantly, but fairly predictably. *Acacia longifolia* invades the total range of microsites in this system, additionally influencing the native vegetation. An earlier study showed that δ^15^N enrichment of *C. album* in the surrounding of *A. longifolia* canopies indicates input of N with an isotopic signature close to 0‰ that originates from symbiotic fixation of atmospheric N_2_^21^. Such an enrichment with N and alterations of nutrient pools and cycling^49–51^ has cascading effects on the recipient community, modifying soil microbial communities^52^ and negatively affecting native seedling diversity^53, 54^. For *A. longifolia*, being a strong competitor for water^55^ and nutrients^56^, theses alterations likely function as a self-facilitation mechanism, with *A. longifolia* showing a greater responsiveness to N addition compared to native species^57, 58^. Furthermore, the increase in soil N has persistent effects even after eradication and may create a positive feed-back loop promoting secondary invasions^59^.

The spatial influence of N-addition extends beyond the canopy by several meters, as revealed by an isoscapes approach^12, 21, 30^. However, the suitability of δ^15^N as a reliable tracer of N sources in heterogeneous natural ecosystems has been questioned due to the multitude of processes affecting δ^15^N^25, 39^. Here we show that the effect of N input by *A. longifolia* can be disentangled from further controls of δ^15^N in the native system, by accounting for topographic attributes and vegetation cover in a generalized additive mixed model that allows modelling multiple effects that may be highly nonlinear. Therefore, by enabling inferences and predictions of plant functional attributes on a spatially explicit scale, such models can illustrate the dependence of spatial patterns in plant-plant interactions on heterogeneous conditions. In the case of *A. longifolia* invasion, the model can be useful to evaluate the susceptibility of different microsites to invader influence and to identify priority sites for management. For example, we found much stronger effects where *A. longifolia* was located higher, i.e. upslope of the native vegetation (Fig. 3c), indicating that management of individuals or stands of the invader at these topographic positions should be prioritized.

The pronounced spatial heterogeneity of δ^15^N that we found within sites challenges the concept of high or low “invasibility” of specific systems^15^. Similar to the fluctuating resource hypothesis^14^ and the concept of niche opportunities^16^, which basically predict spatio-temporal patterns of invasion, e.g., due to disturbance, invasibility as well as invader impact must be expected to vary on small spatial scales even in the absence of disturbance^60^. Moreover, synergistic effects between fluctuations in resources availability and invader resource use can amplify invader impact and result into cascading effects on native vegetation^61^. Such interdependence between invader success or impact and the heterogeneous environment adds another dimension to the recently proposed concept of ‘invasion syndromes’^62^, which points out the significance of context dependency and species × ecosystem interactions for invasion dynamics^62, 63^.

Our geospatial method is suitable to integrate processes across scales, from local interaction neighbourhood to community and even landscape scales. Thus it can provide a framework that is needed to test the effect of heterogeneity on invasibility as well as to quantify invader-driven heterogeneity, including feedbacks between ecosystem engineers and spatially structured habitats.

Therefore, studies which combine ecophysiological indicators with remote sensing provide new opportunities to develop and test spatially explicit theory and models. Remotely sensed information is increasingly available at higher spatial resolution and current research continues to establish links between remote sensing products and ecological and physiological processes (e.g. refs 64–67). It has even been shown that foliar δ^15^N can be resolved with spectroscopic field measurements^68^. By integrating LiDAR-derived information on surface features with stable isotopes as an ecological tracer, our study strengthens this line of research. Moreover, it clearly indicates that spatial variation should be considered as an important factor at the community scale, when studying plant-plant interactions and specifically invader impacts.

In summary, predictive isoscapes, after careful validation, serve to identify controls of spatial variation and to map heterogeneity in ecological processes as well as to quantify and predict the spatial influence of invasive species. Including spatially explicit information, e.g. on topography and vegetation cover, will improve inferences from and predictions of isoscapes. Moreover, linking remote sensing techniques with tracers of biological processes will facilitate and advance the inclusion of spatial processes in ecological theory from small to large spatial scales.

## Methods

### Field Site

We sampled at five sites along a coastal strip of ca. 30 km length at the Atlantic coast of Southwest Portugal (38.18°N, 8.78°W), between the towns of Comporta and Sínes. Climate is Mediterranean, with dry summers and moist winters. Soils are arenosols (FAO classification) with poor water retention capacity and low nutrient availability (see ref. 54). Vegetation cover is low and typically consists of a diverse community of small dwarf shrubs, including *Corema album* (L.) D. Don (Ericaceae), and trees. The area is invaded by the exotic N_2_-fixing *Acacia longifolia* (Andrews) Willd. (Fabaceae), a large shrub or small tree, which forms extensive, very dense monospecific thickets in the dune system^53^.

*C. album* is an endemic species in the sand dune ecosystems of the Iberian Peninsula, which is well-adapted to the nutrient-poor conditions. Foliar nitrogen content of *C. album* is typically very low (e.g. refs 54 and 69), with extraordinarily depleted δ^15^N values that are characteristic for non-fixing species in this strongly N-limited system^30^ and in particular for the ericacean *C. album* ^12, 21, 54^. Therefore, *C. album* has proven a suitable indicator species to measure the impact of N input by the N_2_-fixing invasive *A*. *longifolia* ^12, 21, 54^.

### Leaf sampling

At the five sites, in total eight plots were established. Each plot contained one central *A. longifolia* canopy consisting of one and sometimes two to several inseparable *A. longifolia* individuals, from which four transects of ca. 20 m length were laid out in the four cardinal directions. Along each transect, current year, fully expanded, healthy sunlit leaves of on average 13 ± 3 (mean ± SD) *C. album* plants were sampled in August 2013. In one plot, only three transects could be sampled because of a lack of *C. album* individuals in one direction. Overall, 450 plants were sampled. Additionally, phyllodes of the central *A. longifolia* and of any other large individual in the surrounding were collected. Locations of *A. longifolia* canopies (polygons) and *C. album* plants (points) were recorded using a differential GPS (GeoXH, with a Tornado GA810 Antenna, Trimble, Sunnyvale, CA, USA). The GPS positions were post-processed using reference data from the closest permanent reference station of the Portuguese Direção-Geral do Território in Santiago do Cacém, Portugal. 95% of the measured points were corrected using the GPS carrier phase with an average accuracy of 0.12 m ± 0.08 m. The remaining points were corrected using the code phase (4% of the points, 0.60 m ± 0.49 m) or SBAS (Satellite-based Augmentation System) real time correction (1% of the points, 0.58 m ± 0.10 m).

### Isotope analysis

Samples were oven-dried at 65 °C for at least 48 hours and ground to a fine powder using a ball-mill (Retsch, Haan, Germany). Nitrogen isotope ratios were analysed in an Elemental Analyser (HEKAtech GmbH, Weinberg, Germany) coupled to a continuous flow stable isotope ratio mass spectrometer (ISOPRIME, Elementar, Hanau, Germany). Measurements were related to IAEA-N-1 and IAEA-N-2 standards (International Atomic Energy Agency, Vienna, Austria) and a laboratory standard (IVA33802156, IVA Analysetechnik e.K., Meerbusch, Germany). Isotopic values are expressed in δ notation referenced to the international IAEA standard of air. Repeated measurement precision was 0.2‰.

### LiDAR products

LiDAR (Light Detection And Ranging) data of the study area were acquired with a Leica ALS50-II (Leica Geosystems AG, Heerbrugg, Switzerland) in a flight campaign on April 7, 2011. Average point density was 4.5 points × m^−2^ with an average point spacing of 0.64 m. Multiple Pulse in Air (MPiA) was up to 4 returns. The LiDAR point cloud was scanned for noisy data and ground points were identified to produce a digital elevation model (DEM) with a cell resolution of 1 × 1 m using LAStools (version 141013, rapidlasso GmbH, Gilching, Germany). Relative vegetation cover was computed as the ratio of non-ground points to ground points and filtered with a six cell radius.

### Spatially explicit predictors

To model δ^15^N of *C. album*, raster maps of six spatially explicit predictors were prepared for each study site. As N input by *A. longifolia* has been found to influence δ^15^N values of *C. album*, a raster map containing the distance of each cell to the closest *A. longifolia* canopy larger than 6 m^2^ was created. Additionally, as larger *A. longifolia* canopies are assumed to have a larger influence, raster maps specifying the area of the closest *A. longifolia* canopy were generated. The vertical position of *C. album* relative to *A. longifolia* might further modify its effect on δ^15^N. Therefore, the elevation difference to the closest *A. longifolia* canopy was calculated for each raster cell.

To account for possible effects of soil moisture, the topographic wetness index (TWI) was calculated in SAGA GIS 2.2.3^70^ as ln(*a*/tan*β*), where *a* is the cumulative upslope area draining through a cell per unit contour length, and *β* is the local slope^71^. The topographic wetness index has previously been shown to correlate with soil moisture in a coastal dune field^72^. In SAGA GIS, we ran the TWI toolchain using a Multiple Flow Direction model and the resulting raster was smoothed with a mean filter using a four cell radius to reduce noise.

Furthermore, as the topographic position of a plant could influence its δ^15^N, a landform classification was performed based on a topographic position index (TPI) according to Weiss^73^. The TPI is computed as the difference between the elevation of each cell and the mean elevation for all cells of a moving circular window centred on the cell of interest^74^. Thus, the TPI can be used to identify landforms within a local neighbourhood, i.e., positive values indicate that the cell is higher than its surroundings and negative values mean that it is lower, while values close to 0 can indicate either a plain or a mid-slope position. The landform classification^73^ uses the slope to distinguish between plains and mid-slope positions. The TPI was calculated in SAGA GIS 2.2.3 on the smoothed digital elevation model (mean filter, five cell radius) and using a minimum radius of 10 m and a maximum radius of 100 m. Subsequently, several of the original classes proposed by Weiss^73^ were merged, as some classes had few members or values were similar due to the relatively small spatial extent. The final classes were: ridges, slopes, plains, drainages and valleys (containing valleys and “streams”).

The size of the raster images at each study site was chosen such that they covered a buffer of 100 m radius surrounding the outmost individuals mapped at each site, which resulted in raster images of ca. 6.1–14.8 ha. Spatial resolution was 1 m, according to the resolution of the LiDAR-derived digital elevation model.

From the above raster images, values were extracted at the locations of each *C. album* individual from which foliage was sampled. This dataset was then used to fit a model of foliar δ^15^N of *C. album*. For the area of the closest *A. longifolia* canopy and for vegetation cover, the logarithm was used to approximate normal distributions.

### Statistical analysis

Foliar δ^15^N of *C. album* was modelled using a generalized additive mixed model (GAMM). Generalized additive models provide a flexible statistical means to identify and characterize non-linear regression effects by replacing some or all of the linear terms of the ordinary generalized models by non-parametric smoothing functions^75^. Generalized additive mixed models can additionally handle random effects^76^.

The generalized additive mixed model was fitted in R 3.3.0^77^ with the gamm4 package^78^, using the Gaussian family with identity link function and thin-plate spline smooths. Fixed factors of the model were the parametric term landform class (5 levels) and smoothers for the following continuous variables: log(vegetation cover +1), distance to the closest *A. longifolia* canopy, log(area of the closest *A. longifolia* canopy), elevation relative to the target *A. longifolia* and TWI. Random intercepts were included for each transect nested in the eight plots. Models were fitted with maximum likelihood estimation. To assess model accuracy, the model was run 100 times, each time randomly assigning 75% of the data to a training set and 25% to a test set. The fit of predicted vs. measured values for the test sets was evaluated. A final model was then fitted using all samples. Normal distribution and homoscedasticity of the residuals was verified visually with diagnostic plots. The absence of spatial autocorrelation of the model residuals was confirmed by computing experimental semivariograms and Moran’s I correlograms (Supplementary Figs S6, S7). The relevance of the model terms was assessed in a model selection procedure by minimizing the AIC_c_ (Akaike Information Criterion with finite sample correction) using the function *dredge* from the R package MuMIn^79^. Finally, predictive δ^15^N isoscapes based on the spatially continuous raster maps of the predictors were created at the five sampled sites. Predictions were performed in R using the *predict* function of the raster package^80^ and were restricted to the maximum distance to *A. longifolia* in the training data.

### Data availability statement

The datasets generated during and/or analysed during the current study are available in the figshare repository, 10.6084/m9.figshare.4959881.

## Electronic supplementary material

supplementary information

## References

[1] Tilman D (1987). On the meaning of competition and the mechanisms of competitive superiority. Funct. Ecol..

[2] Tamme R, Hiiesalu I, Laanisto L, Szava-Kovats R, Partel M (2010). Environmental heterogeneity, species diversity and co-existence at different spatial scales. J. Veg. Sci..

[3] Stein A, Gerstner K, Kreft H (2014). Environmental heterogeneity as a universal driver of species richness across taxa, biomes and spatial scales. Ecol. Lett..

[4] Wardle DA (2004). Ecological linkages between aboveground and belowground biota. Science.

[5] Ehrenfeld JG, Ravit B, Elgersma K (2005). Feedback in the plant-soil system. Annu. Rev. Environ. Resour..

[6] Callaway RM, Walker LR (1997). Competition and facilitation: A synthetic approach to interactions in plant communities. Ecology.

[7] Bertness MD, Callaway R (1994). Positive interactions in communities. Trends Ecol. Evol..

[8] Maestre FT, Callaway RM, Valladares F, Lortie CJ (2009). Refining the stress-gradient hypothesis for competition and facilitation in plant communities. J. Ecol.

[9] Soliveres S, Smit C, Maestre FT (2015). Moving forward on facilitation research: response to changing environments and effects on the diversity, functioning and evolution of plant communities. Biol. Rev..

[10] Dickie IA, Schnitzer SA, Reich PB, Hobbie SE (2005). Spatially disjunct effects of co-occurring competition and facilitation. Ecol. Lett..

[11] Berger U, Piou C, Schiffers K, Grimm V (2008). Competition among plants: Concepts, individual-based modelling approaches, and a proposal for a future research strategy. Perspect. Plant Ecol. Evol. Syst..

[12] Hellmann C, Rascher KG, Oldeland J, Werner C (2016). Isoscapes resolve species-specific spatial patterns in plant–plant interactions in an invaded Mediterranean dune ecosystem. Tree Physiol..

[13] Hobbs RJ, Huenneke LF (1992). Disturbance, diversity, and invasion: Implications for conservation. Conserv. Biol..

[14] Davis MA, Grime JP, Thompson K (2000). Fluctuating resources in plant communities: a general theory of invasibility. J. Ecol..

[15] Lonsdale WM (1999). Global patterns of plant invasions and the concept of invasibility. Ecology.

[16] Shea K, Chesson P (2002). Community ecology theory as a framework for biological invasions. Trends Ecol. Evol..

[17] Dawson TE, Mambelli S, Plamboeck AH, Templer PH, Tu KP (2002). Stable isotopes in plant ecology. Annu. Rev. Ecol. Syst..

[18] West JB, Bowen GJ, Cerling TE, Ehleringer JR (2006). Stable isotopes as one of nature’s ecological recorders. Trends Ecol. Evol..

[19] Werner C (2012). Progress and challenges in using stable isotopes to trace plant carbon and water relations across scales. Biogeosciences.

[20] West JB, Sobek A, Ehleringer JR (2008). A simplified GIS approach to modeling global leaf water isoscapes. PLoS ONE.

[21] Rascher KG, Hellmann C, Máguas C, Werner C (2012). Community scale ^15^N isoscapes: tracing the spatial impact of an exotic N_2_-fixing invader. Ecol. Lett.

[22] Bai E, Boutton TW, Liu F, Wu XB, Archer SR (2013). ^15^N isoscapes in a subtropical savanna parkland: spatial-temporal perspectives. Ecosphere.

[23] Nitzsche KN, Verch G, Premke K, Gessler A, Kayler ZE (2016). Visualizing land-use and management complexity within biogeochemical cycles of an agricultural landscape. Ecosphere.

[24] Högberg P (1997). Tansley Review No. 95 ^15^N natural abundance in soil-plant systems. New Phytol..

[25] Robinson D (2001). δ^15^N as an integrator of the nitrogen cycle. Trends Ecol. Evol..

[26] Kleinebecker T (2014). Evidence from the real world: ^15^N natural abundances reveal enhanced nitrogen use at high plant diversity in Central European grasslands. J. Ecol..

[27] Handley LL, Raven JA (1992). The use of natural abundance of nitrogen isotopes in plant physiology and ecology. Plant Cell Environ.

[28] Hobbie E, Hobbie J (2008). Natural abundance of ^15^N in nitrogen-limited forests and tundra can estimate nitrogen cycling through mycorrhizal fungi: A review. Ecosystems.

[29] Stahl VM, Beyschlag W, Werner C (2011). Dynamic niche sharing in dry acidic grasslands -a ^15^N-labeling experiment. Plant Soil.

[30] Hellmann C, Werner C, Oldeland J (2016). A spatially explicit dual-isotope approach to map regions of plant-plant interaction after exotic plant invasion. PLOS ONE.

[31] Bowen GJ (2010). Isoscapes: Spatial pattern in isotopic biogeochemistry. Annu. Rev. Earth Planet. Sci..

[32] Pardo, L. H. & Nadelhoffer, K. J. Using nitrogen isotope ratios to assess terrestrial ecosystems at regional and global scales in *Isoscapes* (eds West, J. B., Bowen, G. J., Dawson, T. E. & Tu, K. P.) 221–249 (Springer Netherlands, 2010).

[33] Lefsky MA, Cohen WB, Parker GG, Harding DJ (2002). Lidar remote sensing for ecosystem studies. Bioscience.

[34] Ruiz-Navarro A, Barberá GG, García-Haro J, Albaladejo J (2012). Effect of the spatial resolution on landscape control of soil fertility in a semiarid area. J. Soils Sediments.

[35] Ruiz-Navarro A, Barberá GG, Albaladejo J, Querejeta JI (2016). Plant δ^15^N reflects the high landscape-scale heterogeneity of soil fertility and vegetation productivity in a Mediterranean semiarid ecosystem. New Phytol..

[36] Dawson TE (1993). Hydraulic lift and water-use by plants - implications for water-balance, performance and plant-plant interactions. Oecologia.

[37] Finzi AC, Van Breemen N, Canham CD (1998). Canopy tree–soil interactions within temperate forests: Species effects on soil carbon and nitrogen. Ecol. Appl..

[38] Craine JM (2009). Global patterns of foliar nitrogen isotopes and their relationships with climate, mycorrhizal fungi, foliar nutrient concentrations, and nitrogen availability. New Phytol..

[39] Craine JM (2015). Ecological interpretations of nitrogen isotope ratios of terrestrial plants and soils. Plant Soil.

[40] Sutherland RA, van Kessel C, Farrell RE, Pennock DJ (1993). Landscape-scale variations in plant and soil nitrogen-15 natural abundance. Soil Sci. Soc. Am. J..

[41] Bai E (2009). Spatial variation of the stable nitrogen isotope ratio of woody plants along a topoedaphic gradient in a subtropical savanna. Oecologia.

[42] Mardegan S, Nardoto G, Higuchi N, Moreira M, Martinelli L (2009). Nitrogen availability patterns in white-sand vegetations of Central Brazilian Amazon. Trees - Struct. Funct..

[43] Cheesman AW, Cernusak LA (2016). Isoscapes: a new dimension in community ecology. Tree Physiol..

[44] Facelli JM, Pickett STA (1991). Plant litter: Its dynamics and effects on plant community structure. Bot. Rev..

[45] Hobbie EA, Högberg P (2012). Nitrogen isotopes link mycorrhizal fungi and plants to nitrogen dynamics. New Phytol..

[46] Hobbie EA, Colpaert JV (2003). Nitrogen availability and colonization by mycorrhizal fungi correlate with nitrogen isotope patterns in plants. New Phytol..

[47] Álvarez-Cansino L, Zunzunegui M, Díaz Barradas MC, Esquivias MP (2010). Physiological performance and xylem water isotopic composition underlie gender-specific responses in the dioecious shrub *Corema album*. Physiol. Plant..

[48] Bronick CJ, Lal R (2005). Soil structure and management: a review. Geoderma.

[49] Marchante E, Kjøller A, Struwe S, Freitas H (2008). Short- and long-term impacts of *Acacia longifolia* invasion on the belowground processes of a Mediterranean coastal dune ecosystem. Appl. Soil Ecol..

[50] Marchante E, Kjøller A, Struwe S, Freitas H (2009). Soil recovery after removal of the N_2_-fixing invasive *Acacia longifolia*: consequences for ecosystem restoration. Biol. Invasions.

[51] Ulm, F., Hellmann, C., Cruz, C. & Máguas, C. N/P imbalance as a key driver for the invasion of oligotrophic dune systems by a woody legume. *Oikos*, doi:10.1111/oik.03810 (2016).

[52] Rodríguez-Echeverría S (2010). Rhizobial hitchhikers from Down Under: invasional meltdown in a plant-bacteria mutualism?. J. Biogeogr..

[53] Rascher KG, Große-Stoltenberg A, Máguas C, Meira-Neto J, Werner C (2011). *Acacia longifolia* invasion impacts vegetation structure and regeneration dynamics in open dunes and pine forests. Biol. Invasions.

[54] Hellmann C (2011). Impact of an exotic N_2_-fixing *Acacia* on composition and N status of a native Mediterranean community. Acta Oecologica.

[55] Rascher KG, Máguas C, Große-Stoltenberg A, Werner C (2011). Understory invasion of *A. longifolia* in a Mediterranean pine forest negatively affects the water use and carbon assimilation rates of native trees. Ecosystems.

[56] Werner C, Zumkier U, Beyschlag W, Máguas C (2010). High competitiveness of a resource demanding invasive acacia under low resource supply. Plant Ecol..

[57] Peperkorn R, Werner C, Beyschlag W (2005). Phenotypic plasticity of an invasive acacia versus two native Mediterranean species. Funct. Plant Biol..

[58] Werner, C., Peperkorn, R., Máguas, C. & Beyschlag, W. Competitive balance between the alien invasive Acacia longifolia and native Mediterranean species in *Plant Invasions: Human perception, ecological impacts and management* (eds Tokarska-Guzik, B. *et al*.) 261–275 (Backhuys Publishers, 2008).

[59] Marchante H, Marchante E, Freitas H, Hoffmann JH (2015). Temporal changes in the impacts on plant communities of an invasive alien tree. Acacia longifolia. Plant Ecol..

[60] Melbourne BA (2007). Invasion in a heterogeneous world: resistance, coexistence or hostile takeover?. Ecol. Lett..

[61] Caldeira MC (2015). Synergy of extreme drought and shrub invasion reduce ecosystem functioning and resilience in water-limited climates. Sci. Rep.

[62] Kueffer C, Pyšek P, Richardson DM (2013). Integrative invasion science: model systems, multi-site studies, focused meta-analysis and invasion syndromes. New Phytol..

[63] Thiele J, Kollmann J, Markussen B, Otte A (2010). Impact assessment revisited: improving the theoretical basis for management of invasive alien species. Biol. Invasions.

[64] Sims DA, Gamon JA (2002). Relationships between leaf pigment content and spectral reflectance across a wide range of species, leaf structures and developmental stages. Remote Sens. Environ..

[65] Dobrowski SZ, Pushnik JC, Zarco-Tejada PJ, Ustin SL (2005). Simple reflectance indices track heat and water stress-induced changes in steady-state chlorophyll fluorescence at the canopy scale. Remote Sens. Environ..

[66] Porcar-Castell A (2014). Linking chlorophyll a fluorescence to photosynthesis for remote sensing applications: mechanisms and challenges. J. Exp. Bot..

[67] Große-Stoltenberg A, Hellmann C, Werner C, Oldeland J, Thiele J (2016). Evaluation of continuous VNIR-SWIR spectra versus narrowband hyperspectral indices to discriminate the invasive *Acacia longifolia* within a Mediterranean dune ecosystem. Remote Sens..

[68] Hellmann C, Große-Stoltenberg A, Lauströer V, Oldeland J, Werner C (2015). Retrieving nitrogen isotopic signatures from fresh leaf reflectance spectra: disentangling δ^15^N from biochemical and structural leaf properties. Front. Plant Sci..

[69] Álvarez-Cansino L, Barradas MCD, Zunzunegui M, Esquivias MP, Dawson TE (2012). Gender-specific variation in physiology in the dioecious shrub *Corema album* throughout its distributional range. Funct. Plant Biol..

[70] Conrad O (2015). System for Automated Geoscientific Analyses (SAGA) v. 2.1.4. Geosci. Model Dev.

[71] Beven KJ, Kirkby MJ (1979). A physically based, variable contributing area model of basin hydrology. Hydrol. Sci. J..

[72] Kim D, Yu KB (2008). A conceptual model of coastal dune ecology synthesizing spatial gradients of vegetation, soil, and geomorphology. Plant Ecol..

[73] Weiss, A. D. Topographic position and landforms analysis (2000). Available at: www.jennessent.com/downloads/tpi-poster-tnc_18x22.pdf (Accessed: 1st September 2016).

[74] Guisan A, Weiss SB, Weiss AD (1999). GLM versus CCA spatial modeling of plant species distribution. Plant Ecol..

[75] Hastie, T. J. & Tibshirani, R. J. *Generalized additive models* (CRC Press, 1990). 10.1177/096228029500400302 8548102

[76] Lin X, Zhang D (1999). Inference in generalized additive mixed models by using smoothing splines. J. R. Stat. Soc. Ser. B Stat. Methodol..

[77] R Core Team. R: a language and environment for statistical computing. R Foundation for Statistical Computing: Vienna, Austria (2016).

[78] Wood, S. & Scheipl, F. gamm4: Generalized additive mixed models using mgcv and lme4. R package version 0.2–3 (2014).

[79] Barton, K. MuMIn: Multi-Model Inference. R package version 1.15.6 (2016).

[80] Hijmans, R. J. raster: Geographic Data Analysis and Modeling. R package version 2.3–40 (2015).

