# Supplementary material for: Heterogeneous environments shape invader impacts: integrating environmental, structural and functional effects by isoscapes and remote sensing
Source: Sci Rep. 2017 Jun 23;7:4118. doi: 10.1038/s41598-017-04480-4 (PMC5482842; doi:10.1038/s41598-017-04480-4)
Supplement: Supplementary file 1 — supplementary information [file 41598_2017_4480_MOESM1_ESM.pdf]

Supplementary Information for

Heterogeneous environments shape invader impacts: integrating environmental, structural and functional effects by isoscapes and remote sensing

Christine Hellmann[1,2], André Große-Stoltenberg[3], Jan Thiele[3], Jens Oldeland[4], Christiane Werner[1]*

[1] *Ecosystem Physiology, University of Freiburg, Georges-Köhler-Allee 53/54, 79110 Freiburg, Germany*
[2] *Experimental and Systems Ecology, University of Bielefeld, Universitätsstraße 25, 33615 Bielefeld, Germany*
[3] *Institute of Landscape Ecology, University of Münster, Heisenbergstraße 2, 48149 Münster, Germany*
[4] *Biodiversity, Ecology and Evolution of Plants, Biocentre Klein Flottbek and Botanical Garden, University of Hamburg, Ohnhorststraße 18, 22609 Hamburg, Germany*

* Corresponding author

Content

Supplementary Figure S1 | Measured and predicted foliar $\delta^{15}N$ values of *Corema album* along individual transects with increasing distance to the closest Acacia longifolia canopy

Supplementary Figures S2-S5 | Model simulations to illustrate the additive effects of three selected spatially explicit predictors on model estimates for the study sites Praia do Pego, Herdade do Pinheirinho, Aberta Nova and Lagoa da Sancha.

Supplementary Figure S6 | Experimental semivariograms of model residuals

Supplementary Figure S7 | Moran's I correlograms of model residuals

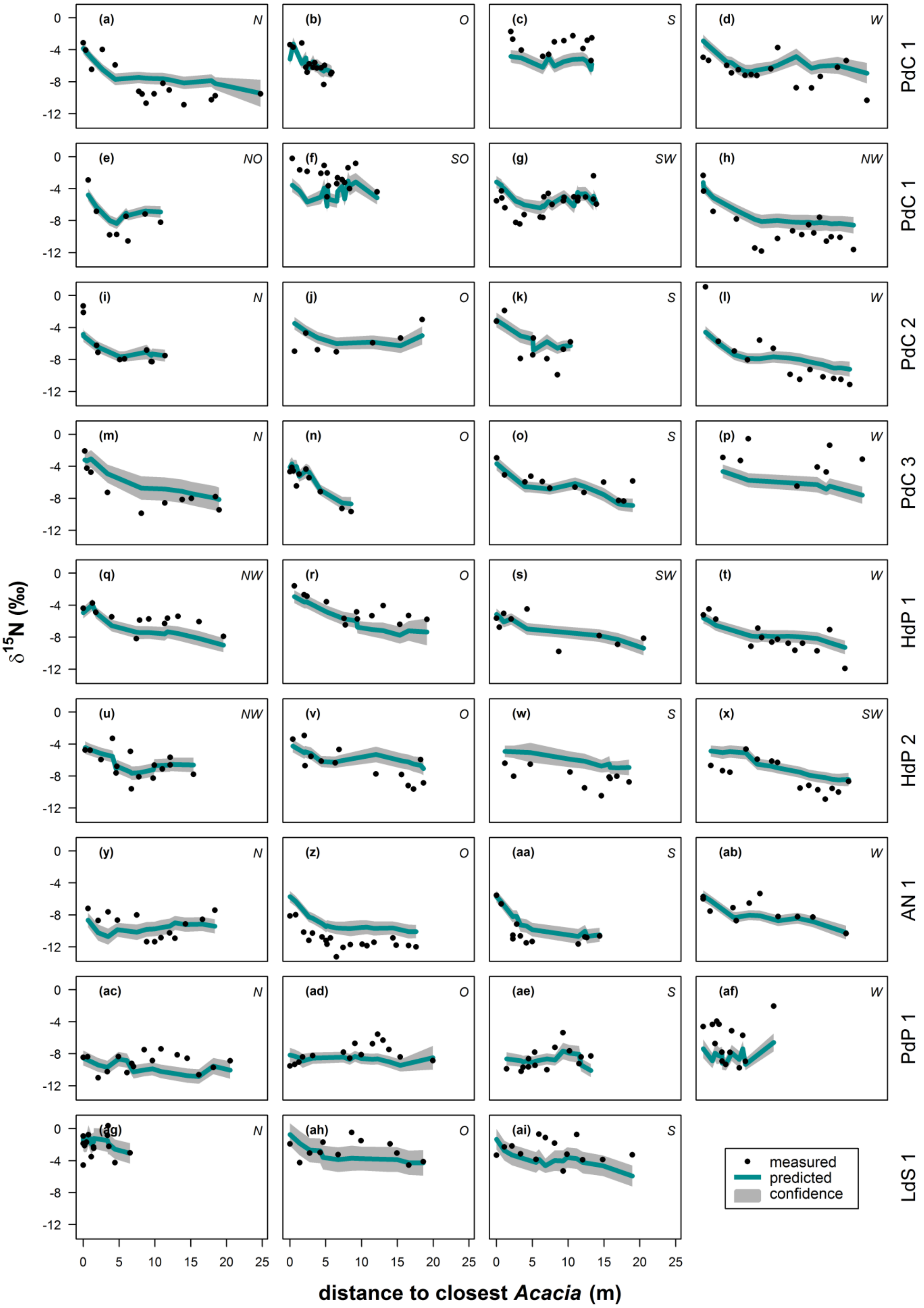

Supplementary Figure S1. Measured foliar $\delta^{15}N$ values of *Corema album* (points) along with the predicted values (lines) and the 95% confidence intervals (grey shading). Individual transects are shown, displaying $\delta^{15}N$ as a function of the distance to the closest *Acacia longifolia* canopy. Cardinal directions are indicated by italic capital letters in the upper right corners. Random effects were not included in the predictions. Each row presents one plot, with locations and plot numbers specified at the right side of the panel. PdC 1 occupies 2 rows, as eight transects were measured in this plot and LdS 1 had only three transects. PdC – Pinheiro da Cruz; HdP – Herdade do Pinheirinho; AN – Aberta Nova; PdP – Praia do Pego; LdS – Lagoa da Sancha.

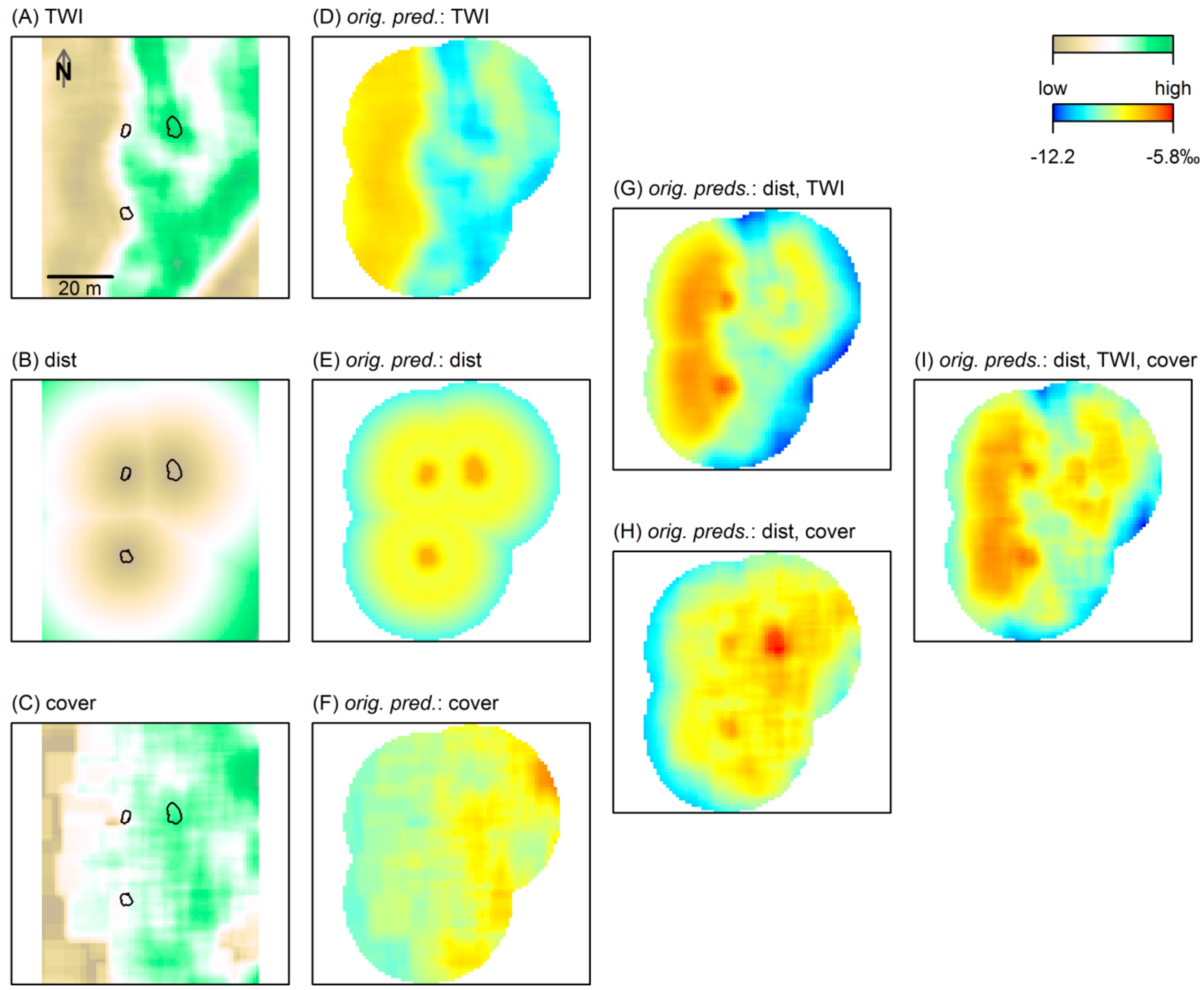

Supplementary Figure S2. Model simulations illustrating the additive effects of three selected spatially explicit predictors on model estimates for the study site Praia do Pego. **(a–c)** Raster maps of the predictors TWI (topographic wetness index), *dist* (distance to the closest *Acacia longifolia* canopy) and *cover* (log(vegetation cover + 1)) are shown. Black outlines demarcate *A. longifolia* canopies. **(d–i)** Model simulations of *Corema album* foliar $\delta^{15}N$ using a generalized additive mixed model (GAMM) with six predictors (including TWI, *dist* and *cover*, see Methods section for details). Predictors specified in the panel heading are included with their original spatial variation while all other predictors are held constant at their mean (for continuous predictors) or at the most frequent class (for factors). First, only one predictor was used with its original variation, with all other variables held constant **(d–f)**, second, additive effects of two original predictors are illustrated **(g–h)**, and finally, **(i)** shows the combined effect when taking into account the spatial variation of three predictors.

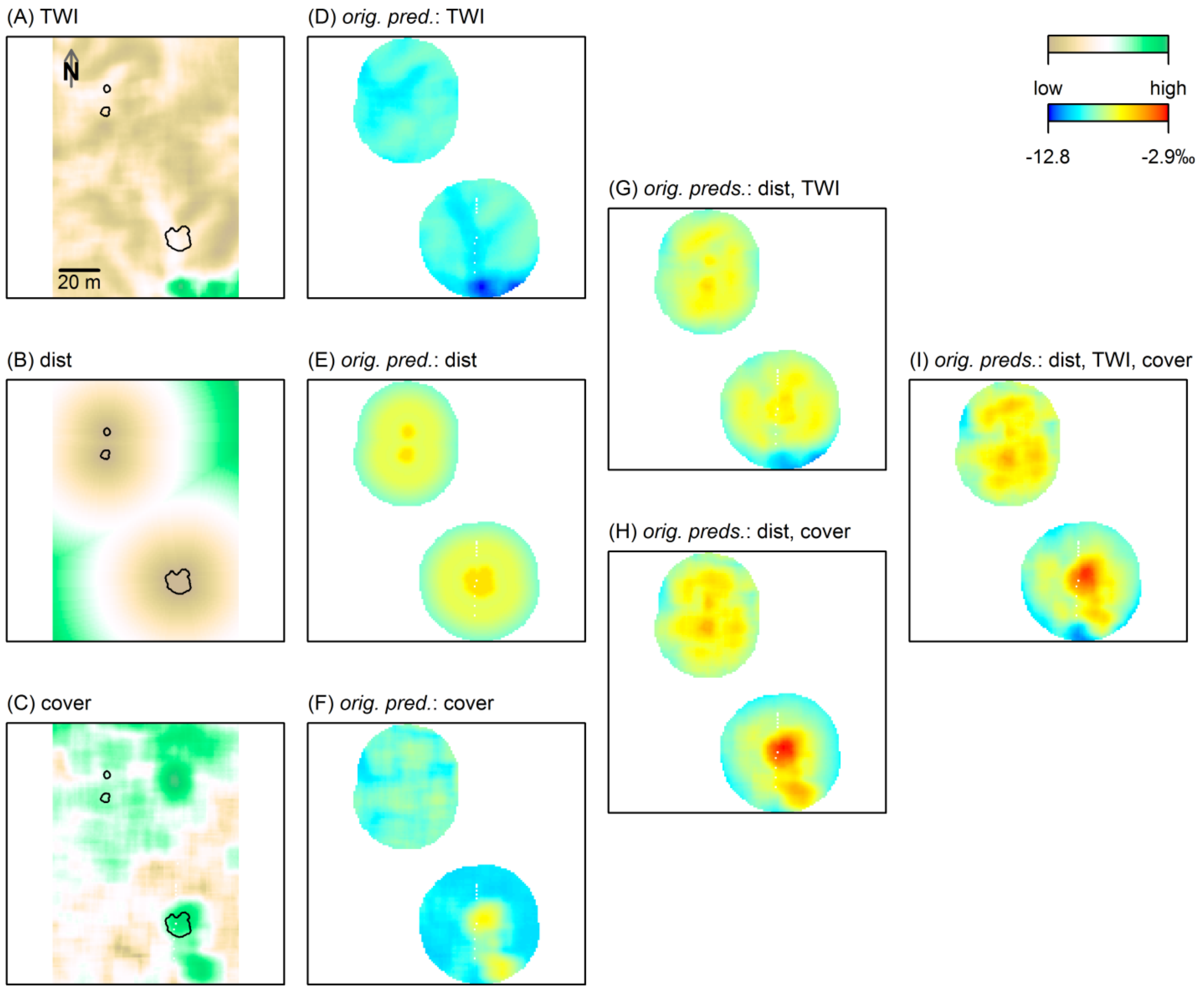

Supplementary Figure S3. Model simulations illustrating the additive effects of three selected spatially explicit predictors on model estimates for the study site Herdade do Pinheirinho. For a detailed description of the panels please see Figure caption of Supplementary Fig. S2.

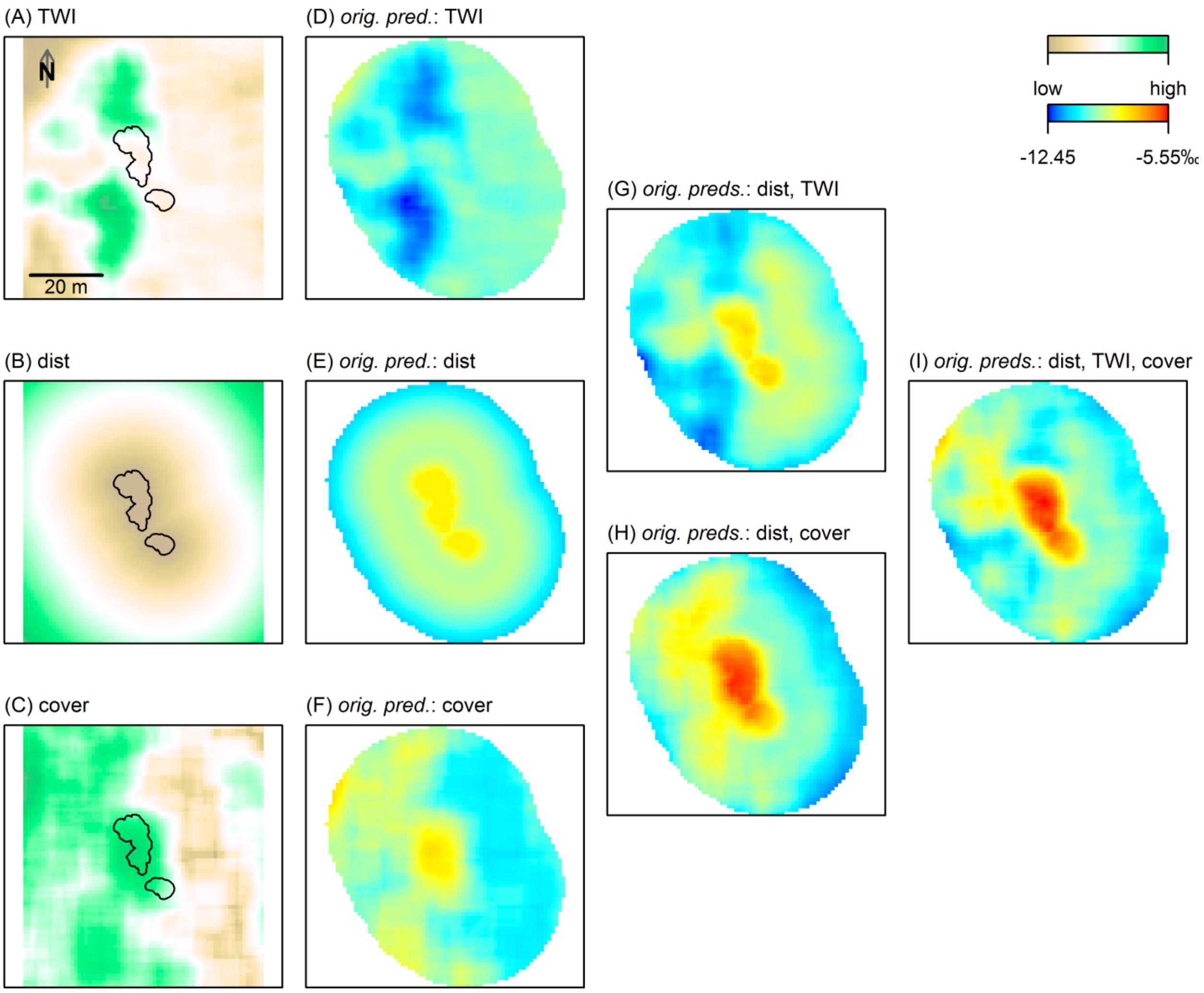

Supplementary Figure S4. Model simulations illustrating the additive effects of three selected spatially explicit predictors on model estimates for the study site Aberta Nova. For a detailed description of the panels please see Figure caption of Supplementary Fig. S2.

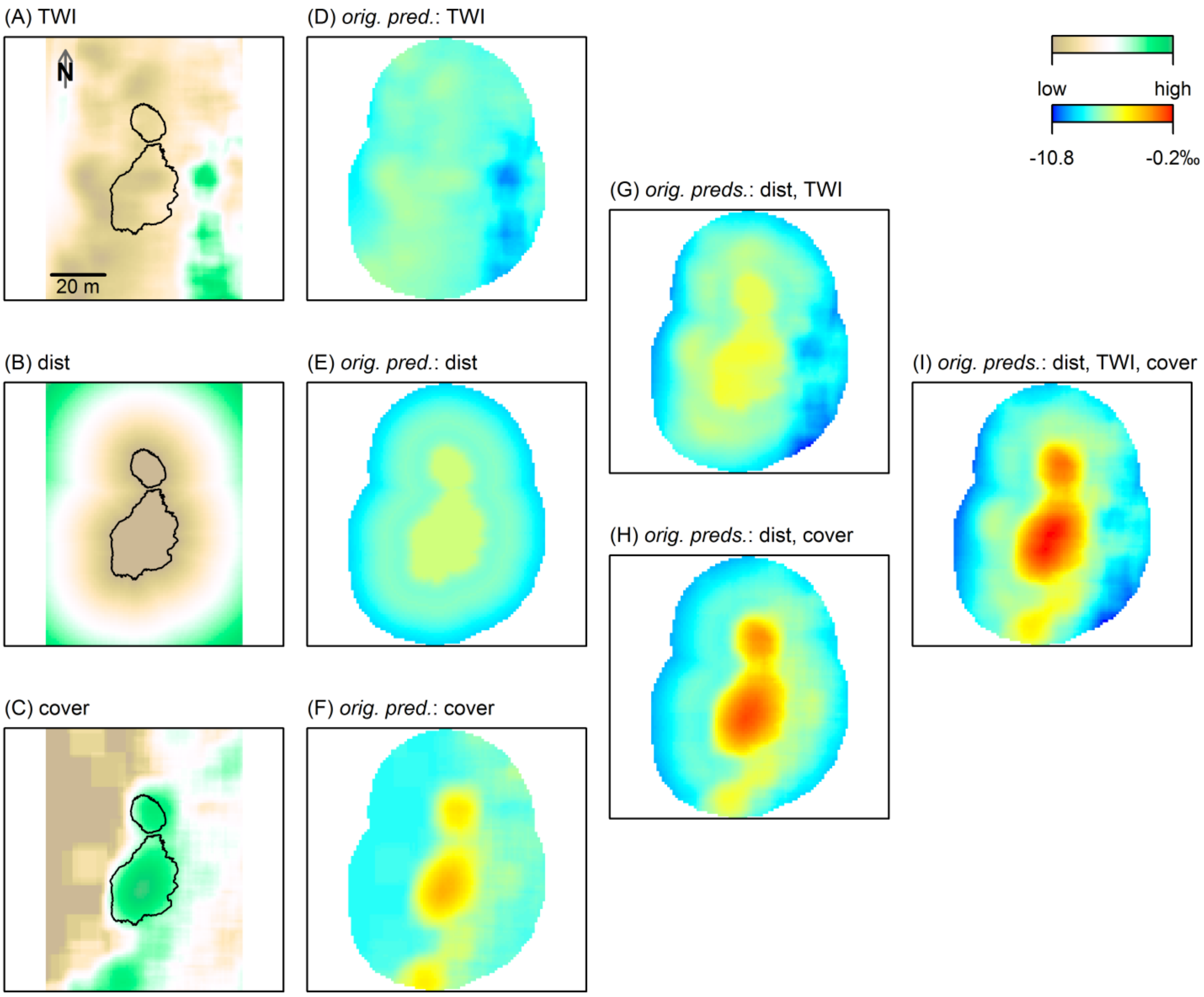

Supplementary Figure S5. Model simulations illustrating the additive effects of three selected spatially explicit predictors on model estimates for the study site Lagoa da Sancha. For a detailed description of the panels please see Figure caption of Supplementary Fig. S2.

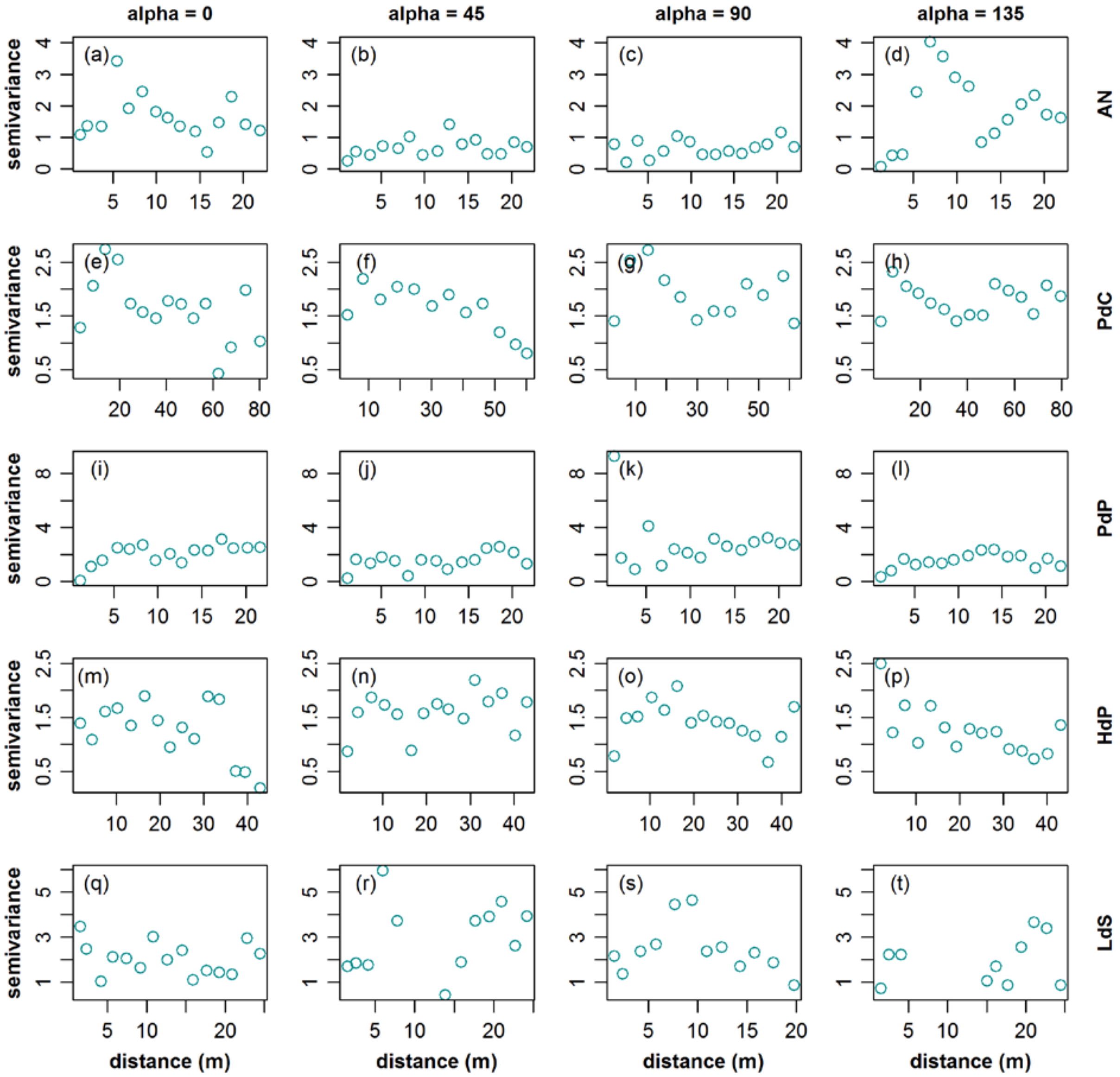

Supplementary Figure S6. Experimental semivariograms of model residuals from the generalized additive mixed model (GAMM) at the five study sites. Semivariograms were sampled in four directions (alpha = 0°, 45°, 90°, 135°), to account for potential anisotropy.
PdC – Pinheiro da Cruz; HdP – Herdade do Pinheirinho; AN – Aberta Nova; PdP – Praia do Pego; LdS – Lagoa da Sancha.

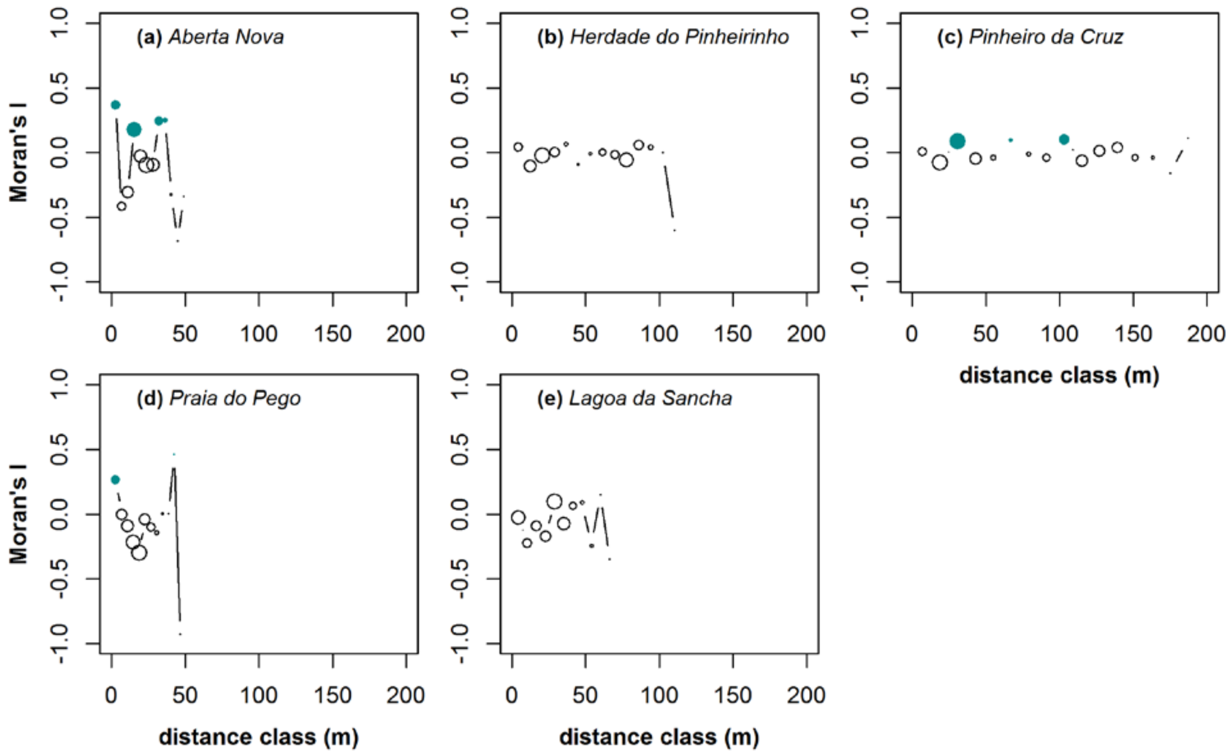

Supplementary Figure S7. Moran's I correlograms of model residuals at the five study sites. Symbol size corresponds to the sample size in the respective distance class. Significant values (normal approximation, two-sided, $P < 0.05$) are shown as closed symbols.
